# Bridging the simulation-to-real gap for AI-based needle and target detection in robot-assisted ultrasound-guided interventions

**DOI:** 10.1186/s41747-023-00344-x

**Published:** 2023-06-19

**Authors:** Visar Arapi, Alexander Hardt-Stremayr, Stephan Weiss, Jan Steinbrener

**Affiliations:** Control of Networked Systems Research Group, Institute of Smart Systems Technologies, University of Klagenfurt, Klagenfurt, Austria

**Keywords:** Calibration, Radiology (interventional), Robotics, Ultrasonography

## Abstract

**Background:**

Artificial intelligence (AI)-powered, robot-assisted, and ultrasound (US)-guided interventional radiology has the potential to increase the efficacy and cost-efficiency of interventional procedures while improving postsurgical outcomes and reducing the burden for medical personnel.

**Methods:**

To overcome the lack of available clinical data needed to train state-of-the-art AI models, we propose a novel approach for generating synthetic ultrasound data from real, clinical preoperative three-dimensional (3D) data of different imaging modalities. With the synthetic data, we trained a deep learning-based detection algorithm for the localization of needle tip and target anatomy in US images. We validated our models on real, *in vitro* US data.

**Results:**

The resulting models generalize well to unseen synthetic data and experimental *in vitro* data making the proposed approach a promising method to create AI-based models for applications of needle and target detection in minimally invasive US-guided procedures. Moreover, we show that by one-time calibration of the US and robot coordinate frames, our tracking algorithm can be used to accurately fine-position the robot in reach of the target based on 2D US images alone.

**Conclusions:**

The proposed data generation approach is sufficient to bridge the simulation-to-real gap and has the potential to overcome data paucity challenges in interventional radiology. The proposed AI-based detection algorithm shows very promising results in terms of accuracy and frame rate.

**Relevance statement:**

This approach can facilitate the development of next-generation AI algorithms for patient anatomy detection and needle tracking in US and their application to robotics.

**Key points:**

• AI-based methods show promise for needle and target detection in US-guided interventions.

• Publicly available, annotated datasets for training AI models are limited.

• Synthetic, clinical-like US data can be generated from magnetic resonance or computed tomography data.

• Models trained with synthetic US data generalize well to real *in vitro* US data.

• Target detection with an AI model can be used for fine positioning of the robot.

**Graphical Abstract:**

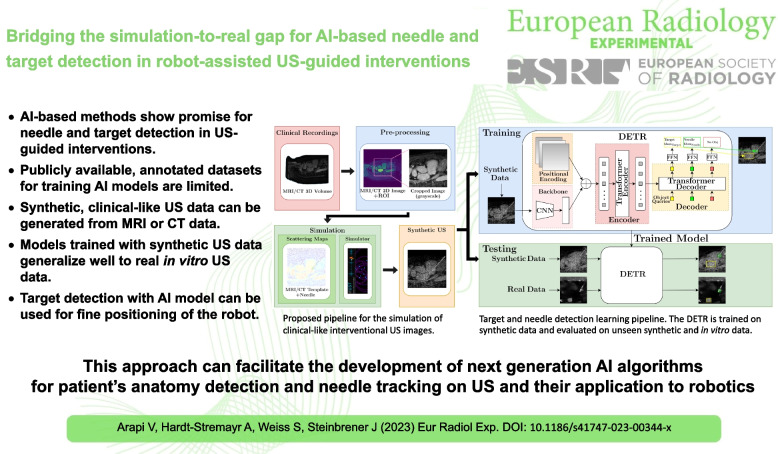

## Background

Interventional radiology has been credited with a variety of benefits such as reduced recovery times, reduced rates and severity of post-surgical complications, higher patient acceptance rates, and higher cost efficiency [1]. Recently, robot-assisted, interventional radiology has been on the rise [2–4]. While many different devices for different applications exist [5], almost all robotic systems available for clinical use today are teleoperators or assistants for holding and aiming [6]. Developing systems that are able to operate at higher autonomy levels even in difficult conditions poses significant research challenges.

Of particular importance for such systems is the capability to continuously track the surgical tool and relevant anatomy during the interventional procedure to handle organ movement and breathing. Due to its cost-efficiency, portability, and absence of radiation damage concerns, ultrasound (US) is an ideal imaging modality for autonomous interventions performed by a robot. Compared to x-ray imaging, the US poses significant challenges for needle and anatomy detection due to its numerous image artifacts.

Several US image processing methods have been proposed to improve needle visibility [7]. Some authors [8–10] used full or partial brightness of the needle in the US image to reconstruct its shape. Other authors [11] introduced a two-phase method based on a needle-specific multi-echo model, showing very good performance but lacking in generalizability. To address this, dynamic intensity changes arising from needle movement in the US image have been used [12, 13].

Recently, approaches based on convolutional neural networks have shown promise for needle detection in static three-dimensional (3D) [14–16] and two-dimensional (2D) US data [17]. Some authors [18] introduced detection transformers for object detection in US images achieving a higher frame rate compared to other state-of-the-art deep learning (DL)-based methods. A major obstacle to developing state-of-the-art AI-based models for analyzing interventional imaging data is the lack of annotated, clinical data for training and testing of the models. While some approaches have been proposed to address data paucity for diagnostic clinical US imaging with the help of generative adversarial networks [19] or through simulation [20], to the best of our knowledge, no work has been proposed so far for the generation of simulation-to-real (sim-to-real) capable training data from simulated US-guided interventions.

We propose here a novel scheme to generate synthetic training data for US-guided, needle-based interventions and validate our approach with *in vitro* data collected from a triple-modality abdominal phantom using the Micromate robot by iSYS Medizintechnik GmbH (Kitzbühel, Austria) [21] with a clinical US system. Our contributions are (i) the development of a simulation pipeline for generating synthetic US training data for needle interventions from preoperative, annotated 3D imaging data of a different imaging modality, *i.e.,* magnetic resonance imaging (MRI) or computed tomography (CT)); (ii) the adaptation of a state-of-the-art DL-based tracking algorithm for US data; (iii) its training and testing with synthetic US data and its deployment on unseen *in vitro* data; and (iv) the illustration of a “robot in reach of target” method for fine positioning of the robot prior to needle insertion based on 2D US images alone.

## Methods

We present a framework for synthetic US data generation based on available annotated CT and MRI datasets for the training and validation of a DL-based needle and lesion-tracking algorithm for use in medical robotics. The annotations consist of 3D target boundary masks and type information (*i.e.,* organs or lesions). Slices of CT/MRI annotated images are combined with artificial needle geometries to generate 2D images for the US simulator. The generated US images are used for the training and validation of the proposed needle and target tracking algorithm. We validated the model with two *in vitro* datasets and show that it can be used to accurately maneuver the robot to the desired body location.

### Experimental setup

**Fig. 1:**
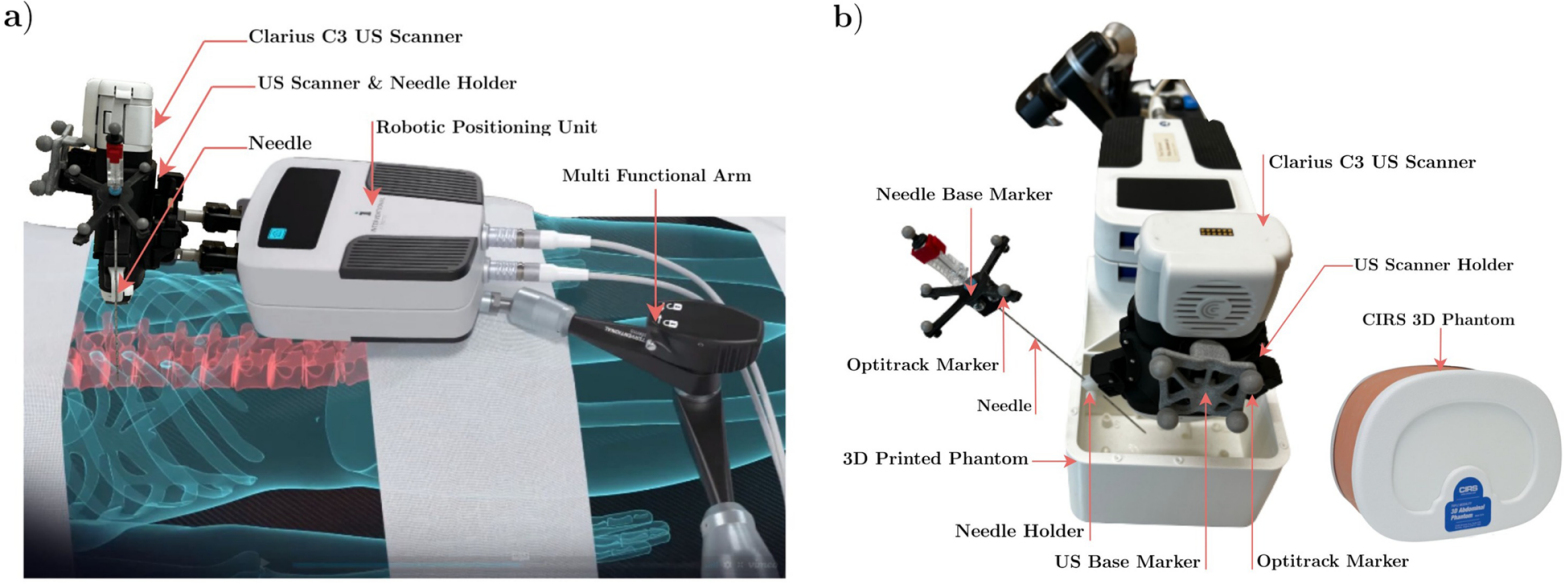
Interventional robotic system adopted in this work. **a** The ultrasound (US) scanner and the needle are mounted directly on the iSYS Micromate™ medical robotic platform through a specially designed holder so that the field of view of the US scanner can be controlled with the robot. **b** Experimental setup used for the *in vitro* US dataset collection. Reflective markers are attached via rigid bases to the US scanner and to the needle posterior allowing their tracking with the infrared camera system

The interventional robotic system adopted in this paper is illustrated in Fig. 1a. The US scanner Clarius C3 (Clarius Mobile Health, Vancouver, Canada) [22] is mounted directly on the iSYS Micromate™ robotic platform (iSYS Medizintechnik GmbH, Kitzbühel, Austria) such that its field of view can be controlled with the robot. The setup includes lateral mounting possibilities for needles with options for five different insertion angles, all coplanar with the imaging plane. The initial positioning of the robot is performed by hand. Then, fine positioning of the US scanner is realized using the robotic stage (4 degrees of freedom). We provide a detailed description of the experimental setup depicted in Fig. 1b.

#### Ultrasound scanner

Because of its high resolution and availability of raw data, we selected the wireless C3 scanner (Clarius Mobile Health, Vancouver, Canada), which has 192 elements and operates at frequencies between 2 and 6 MHz. The viewing angle is 73°, with a penetration depth of up to 400 mm.

#### Motion capture (MoCap) system

We used a MoCap system (8 OptiTrack Cameras Prime 17 W [23], four 9.5-mm markers on US scanner, four 4-mm markers on distal needle end) to collect the ground-truth data for the implementation and evaluation of the “robot in reach of target” algorithm. Using the provided Motive software (NaturalPoint Corporation, Corvallis, USA), we achieve ± 0.2 mm positional accuracy, < 9 ms latency, and ± 0.1° rotational accuracy.

#### Needle

We selected a coated needle (iTP KIR17/20:T, Innovative Tomography Products GmbH, Bochum, Germany) designed for US imaging in biopsy interventions with a diameter of 1.52 mm and a length of 200 mm.

#### CIRS triple modality phantom

We used model 057A (CIRS, Norfolk, VA, USA), which is based on a small adult abdomen and can be scanned with CT, MRI, and US. Multiple biopsy insertions with minimal needle tracking can be executed due to its self-healing capabilities.

#### 3D printed phantom

We printed a technical phantom with embedded fiducial targets (target number 9; mean diameter 8.1 mm, distributed over approximately 130 × 80 × 90 mm^3^) for evaluating aiming accuracy. The phantom was filled with gelatin.

### Dataset creation

**Fig. 2:**
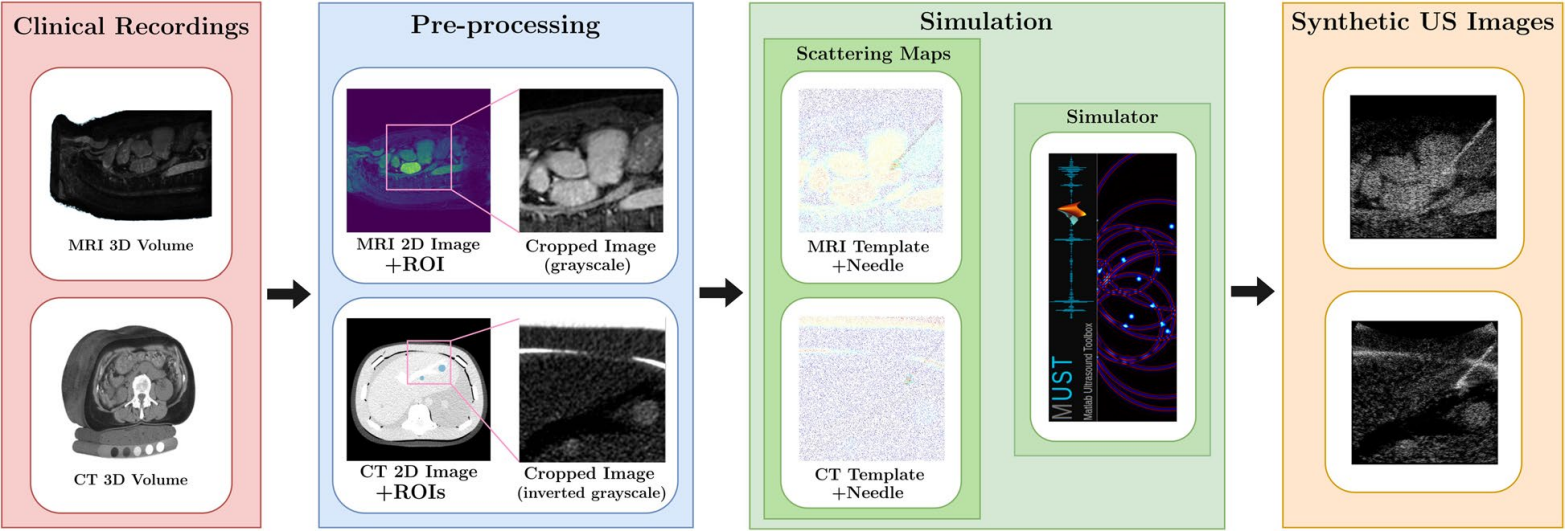
Proposed pipeline for the simulation of clinical-like interventional ultrasound (US) images. Single images are extracted from the three-dimensional magnetic resonance imaging (MRI)/computed tomography (CT) data and preprocessed. The MRI/CT data work as a clinical recording template for speckle texture and anatomy definition. Needle scatterers created artificially are added to the MRI/CT speckle texture. The MUST simulator merging information from the template image and the needle geometry accounts for the US image formation process

We used the pipeline described in Fig. 2 to generate the training dataset for the needle and target detection algorithm. Furthermore, we collected two *in vitro* datasets using the setup depicted in Fig. 1b for the validation of the detection algorithm.

#### Ultrasound simulation

From available numerical US tools [24–26], we used the 2D version of SIMUS [26] in our pipeline due to its efficiency. SIMUS is a backscattered US signal simulator for linear, phased, and convex arrays that are included in the Matlab US toolbox. SIMUS computes the received US signal based on the position and reflection coefficient of each scatterer. The simulation parameters were matched with the specifications of our C3 scanner. The overall simulation pipeline is illustrated in Fig. 2 and contains the following steps.

##### Preoperative clinical recordings

A public 3D MRI clinical, annotated dataset [27], and a 3D CT scan of a CIRS 057A phantom [28] were used as the basis for the simulated US images. The two datasets were representative of different clinical situations concerning the imaging modality of the 3D scan data, and contrast and morphology of the target regions. The MRI dataset comprises monomodal scans of the entire heart collected during a single cardiac phase. The annotated target was the left atrium. Although less relevant for interventional radiology, it served as a challenging benchmark for the detection algorithm due to the heterogeneous morphology of the target and low contrast resolution similar to real, clinical US-guided interventions. The CT dataset was of the same type of phantom we used for *in vitro* experiments. As in our phantom, the liver contained six lesions but placed in different spots. Boundary masks for two of the six lesions were provided with the dataset.

##### Pre-processing

For both 3D datasets, we selected slices where the regions of interest were visible and cropped the slices to a field of view and penetration depth typical for US images.

##### Simulation

The gray-scale image *I*_*gb*_ obtained in the pre-processing step was used to create the background scattering map by randomly extracting *N*_*bscat*_ pixels as scatterers. We empirically selected the scatterer density equal to 6 per square wavelength (*N*_*bscat*_ ≈ 48*,*000). To mimic the tissue echogenicity, the intensities of the *I*_*gb*_ image were used to calculate the reflection coefficients *C*_*b*_ of the scatterers. For the needle simulation, we created geometries that approximate the shape in terms of the length and diameter of the real surgical needle used in our experimental setup. Then, we randomly distributed scatterers in the corresponding geometry with a density of 10 scatterers per square wavelength and with reflection coefficients *C*_*ni*_ ∈ [*max*(*C*_*b*_)*/*4*, max*(*C*_*b*_)]. In order to mimic a real intervention, we first delineated the needle initial position and angle of insertion, then we generated a straight trajectory for the needle to follow. The final scatterer maps were obtained by combining the background *C*_*b*_ and the needle *C*_*n*_ scatterer maps.

##### Synthetic US images

The synthetic radiofrequency signals generated by SIMUS were demodulated to obtain in-phase/quadrature signals. Those signals were beam-formed using a delay-and-sum to obtain B-mode images with a dynamic range in dB. We used three values (25, 30, and 35 dB) to generate images that vary in dynamic range.

#### Simulated scenarios

We simulated the following two scenarios using the aforementioned ultrasound simulation pipeline.

##### Scenario #1: From *in vivo* 3D MRI to ultrasound

This scenario generated synthetic data using a public, annotated, clinical 3D MRI dataset [27] of the heart. We selected 20 fully annotated MRI scans (each from a different patient). For each of them, we extracted the 8 most salient consecutive slices (where the target region of interest, *i.e.,* the left atrium, is well visible) from the volumetric MRI data. To create a dynamic scene, we iterated through the 8 slices while simulating the needle insertion, resulting in 48 scatterer templates for the simulated intervention. Needle insertion angles at 40−60° were simulated. In total, 2,880 synthetic images were generated. Annotations for the left atrium (target) on the synthetic US images were adopted from the MRI dataset and the needle tip was annotated based on its pre-defined trajectory.

##### Scenario #2: From *in vitro* 3D CT to ultrasound

In addition to the clinical MRI data, we used the publicly available CT scan of the triple-modality phantom (CIRS 057A, Norfolk, VA, USA) [28] to generate synthetic data. Fourteen slices where the labeled lesions are visible were used to generate the background for fourteen simulated interventions. Despite the fact that only a single image is used as background for each intervention simulation sequence, the background in the synthetic US images changes from image to image due to the random downsampling of the background scatterers. We used the labels provided with the CT dataset for the target regions and followed the same strategy as in scenario#1 for the needle tip trajectory definition and annotation. Each intervention generates 48 different US synthetic images for a single dynamic range resulting in 2,016 images for the dataset.

#### *In vitro* data acquisitions

To validate the detection algorithm trained on synthetic US images, we collected *in vitro* 2D B-mode images using materials and settings specified in Fig. 1b and for the following two scenarios.

##### Scenario #1: 3D abdominal phantom

In this experiment, the robot with a US scanner (Clarius C3) attached was manually positioned on the CIRS 057A phantom. Fine-positioning using the robot was performed until the target lesion was visible in the US images. Once in position, the needle (iTP KIR17/20:T) was inserted at an in-plane angle of 40° up to a depth of 60 mm. We performed three interventions for a total of 670 US images. The ground-truth lesions and needle tip positions were labeled by hand.

##### Scenario #2: 3D gelatin phantom

Here, we used the same experimental setup as in the previous *in vitro* scenario, but with the dedicated 3D-printed phantom filled with commercial gelatin to simulate tissue. The resulting dataset was used to test the DETR algorithm. The insertion of the needle (iTP KIR17/20:T) was performed at 40, 55, and 60°, respectively. We performed 9 interventions for a total of 1,800 US images. This scenario only contains the needle tip with ground-truth position labeled by hand.

### Needle and target detection algorithm

**Fig. 3:**
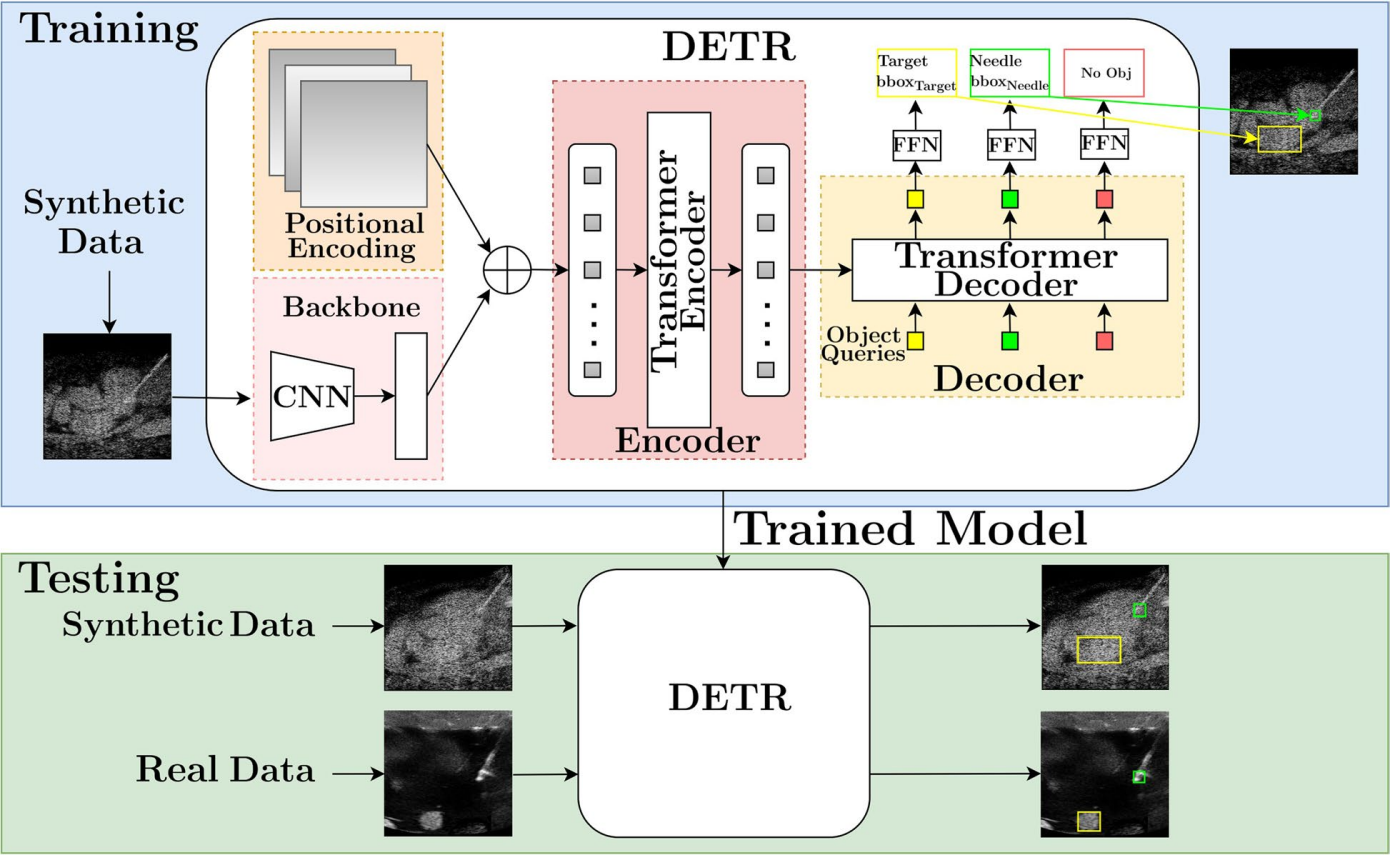
A systematic overview of the target and needle detection learning pipeline. The detection transformer (DETR) is trained on synthetic data and evaluated on unseen synthetic and *in vitro* testing data

To detect the needle and the target in all the aforementioned scenarios, we adapted the state-of-the-art detection transformer (DETR) neural network [29] for US images. As depicted in Fig. 3, DETR uses a convolutional neural network backbone for 2D feature extraction from images. The 2D representation was supplemented with a positional encoding and fed into a transformer encoder. Then, a transformer decoder attends to the encoder output and takes as input a small fixed number of learned positional embeddings (object queries). A shared feed-forward network processes each output embedding of the decoder to classify either a detection (target/needle with bounding box) or a “no object”.

#### Training details of the detection transformer

Since DETR operates on RGB images, we modified the input to consider US gray-scale images and modified its output classes according to ours (“needle” and “target”). The model was initialized from a COCO-pretrained version. AdamW optimizer with an initial learning rate of 10^*-*4^ was used for the fine-tuning. The learning rate and weight decay for the backbone (ResNet-50) were set to 10^*-*5^ and 10^*-*4^, respectively. Xavier initialization was adopted for the weights, and the dropout was set to 0*.*1. We used the same loss function as proposed in literature [29]. The ground-truth needle tip location was the left bottom corner of the bounding box (*x*, *y*), and the size of the bounding box *w* × *h* was chosen to be at least 20 × 20 pixels and at most 30 × 30 pixels. These values were empirically determined in order to ensure the smallest pixel dimensions for an object to be detected by the DETR. For the target annotation, we adopted the original masks with bounding boxes from the annotated MRI/CT datasets. We fine-tuned the DETR on the two simulated datasets using 2,304 (80%) images (scenario #1) and 1,584 (80%) images (scenario #2) for 30 epochs. Both fine-tuned models were tested using the remaining 20% of the simulated datasets and all images of the *in vitro* datasets. Fine-tuning of the DETR took an average of 36 min (scenario #1 dataset) and 27 min (scenario #2 dataset) on a single NVIDIA TITAN RTX 24 GB graphics processing unit. We report the results after 30 epochs of fine-tuning for an overall evaluation. The mean needle and target detection time was 0.03 s which corresponds approximately to 33 frames/s.

### “Robot in reach of target” method

**Fig. 4:**
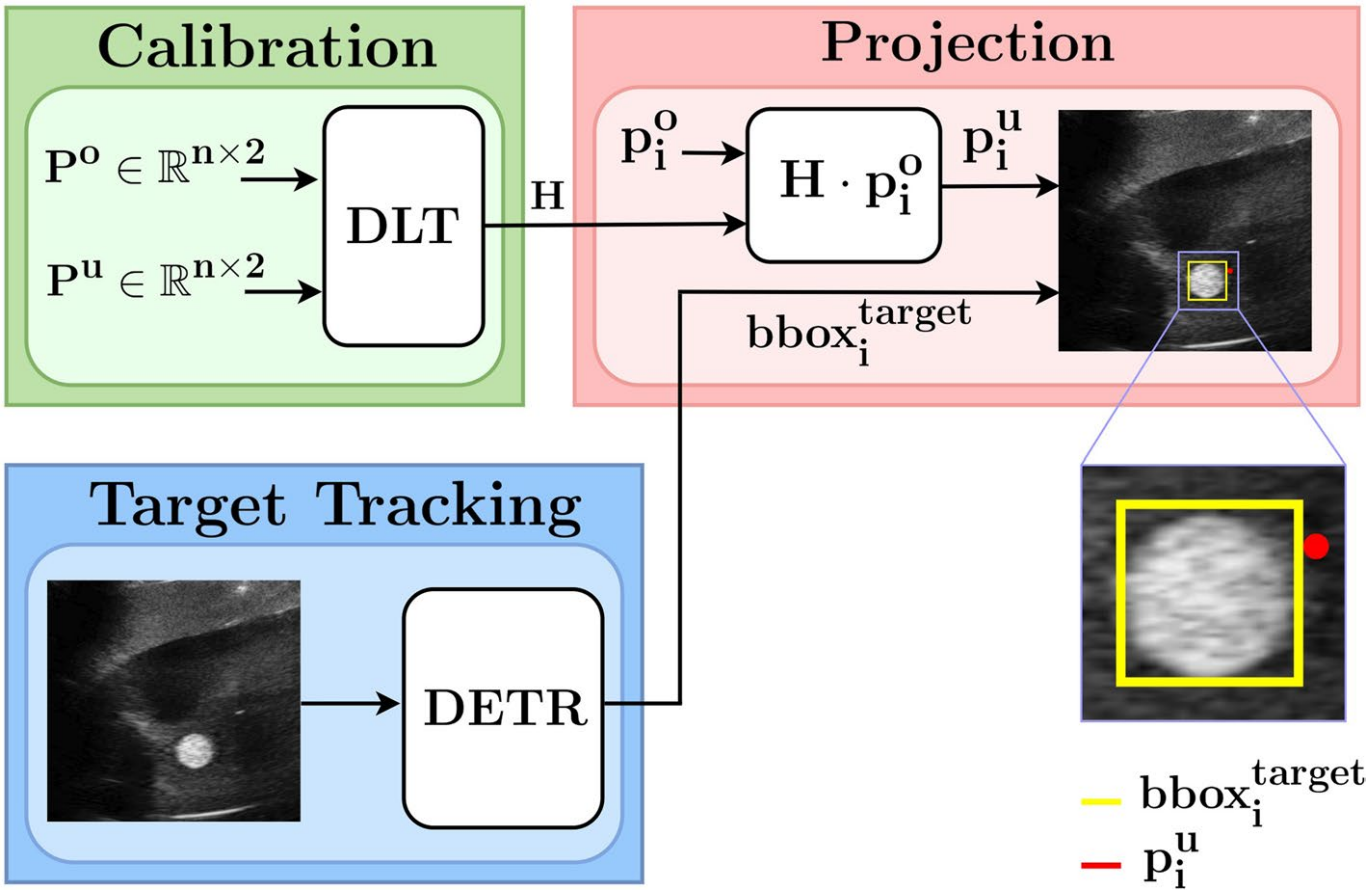
Block diagram used in the robot in reach of the target method. Both the projected point *p*^*u*^ representing the needle tip and the target bounding box provided by the detection transformer (DETR) are exploited for the fine positioning of the needle through the robotic positioning unit and the needle holder

To position the Micromate robot in reach of the target prior to needle insertion using only 2D US images, we make use of our detection algorithm and metric information obtained from an initial, one-time calibration. The latter is performed by tracking the needle tip with our MoCap system in the world frame and simultaneously determining its position in the US images. The calibration procedure determines the transformation from the MoCap (world) frame into the US imaging frame using the direct linear transformation method [30]. The resulting homography matrix *H* can be used to project 2D points from the world frame into the US imaging frame and vice versa. Since the transformation between the US scanner and the needle holder is fixed, an optimal position of the robot with respect to the target can be obtained from US images alone with only the needle in-plane angle as a free parameter. Figure 4 illustrates the overall procedure. Once calibrated, the point p^*u*^ representing the desired needle tip position in the US frame (based, *e.g.,* on the detected target location) can be projected to the external coordinate frame through the homography matrix *H*. This yields the target position for the needle tip p^*o*^ in the external frame. Now the robot can be positioned so that the needle tip is able to reach the desired target location.

## Results

### Needle and target detection results

Tables 1 and 2 show the mean average precision (mAP) of bounding boxes averaged on thresholds ∈ [0*.*5:0*.*05:0*.*95] for all detections and the *total loss* for different testing datasets. Table 1 shows the performance of the model trained on the synthetic scenario#1 dataset. The model trained for detecting both needle and the left atrium (*synth#1-both*) achieved an mAP of 95%, showing a very good detection accuracy. We also trained two additional models, one only for needle detection (*synth#1-needle*) and one only for the left atrium (*synth#1-heart*), respectively. While the former performed very well, achieving an mAP of 98%, the mAP decreased to 80% for the latter. The (*synth#1-needle*) model was evaluated with both *in vitro* datasets for needle tip detection accuracy. The mAPs of both the *in vitro* scenarios (*in-vitro#1-needle*, *in-vitro#2-needle*) were lower (77% and 74%) compared to testing with synthetic data.

**Table 1:** Evaluation of the detection transformer trained on the synthetic heart valve dataset (scenario #1)

Test dataset	Synthetic heart (#1)			*In vitro* CIRS (#1)	*In vitro* phantom (#2)
	Both	Needle	Heart	Needle	Needle
mAP	0.95	0.98	0.8	0.77	0.74
Total loss	2.5	2.1	2.8	3.1	2.9

*mAP* Mean average precision

**Table 2:** Evaluation of the detection transformer trained on the synthetic CIRS (liver) dataset (scenario #2)

Test dataset	Synthetic CIRS (#2)			*In vitro* CIRS (#1)			*In vitro* phantom (#2)
	Both	Needle	Lesion	Both	Needle	Lesion	Needle
mAP	0.97	0.98	0.95	0.83	0.85	0.81	0.86
Total loss	2.1	1.8	2.3	2.8	2.6	3.1	2.7

*mAP* Mean average precision

Table 2 shows the performance of the model trained on the synthetic scenario#2 dataset. The model trained for the detection of the needle tip and the two lesions (*synth#2-both*) achieved an mAP of 97%. The models trained only for needle (*synth#2-needle*) or lesions (*synth#2-lesion*) showed an mAP of 98% and 95%, respectively. The (*synth#2-needle*) model was tested on both *in vitro* datasets. The first *in vitro* scenario was the same as the synthetic scenario#2; hence, we evaluated both the needle tip and the lesions (*in-vitro#1-both*), achieving an mAP of 83%. Then, we individually tested the needle tip detection (*in-vitro#1-needle*) and lesions detection (*in-vitro#1-lesion*), achieving an mAP of 85% and 81%, respectively. Finally, we tested the *in vitro* dataset #2 (*in-vitro#2-needle*) with an mAP of 86%.

**Fig. 5:**
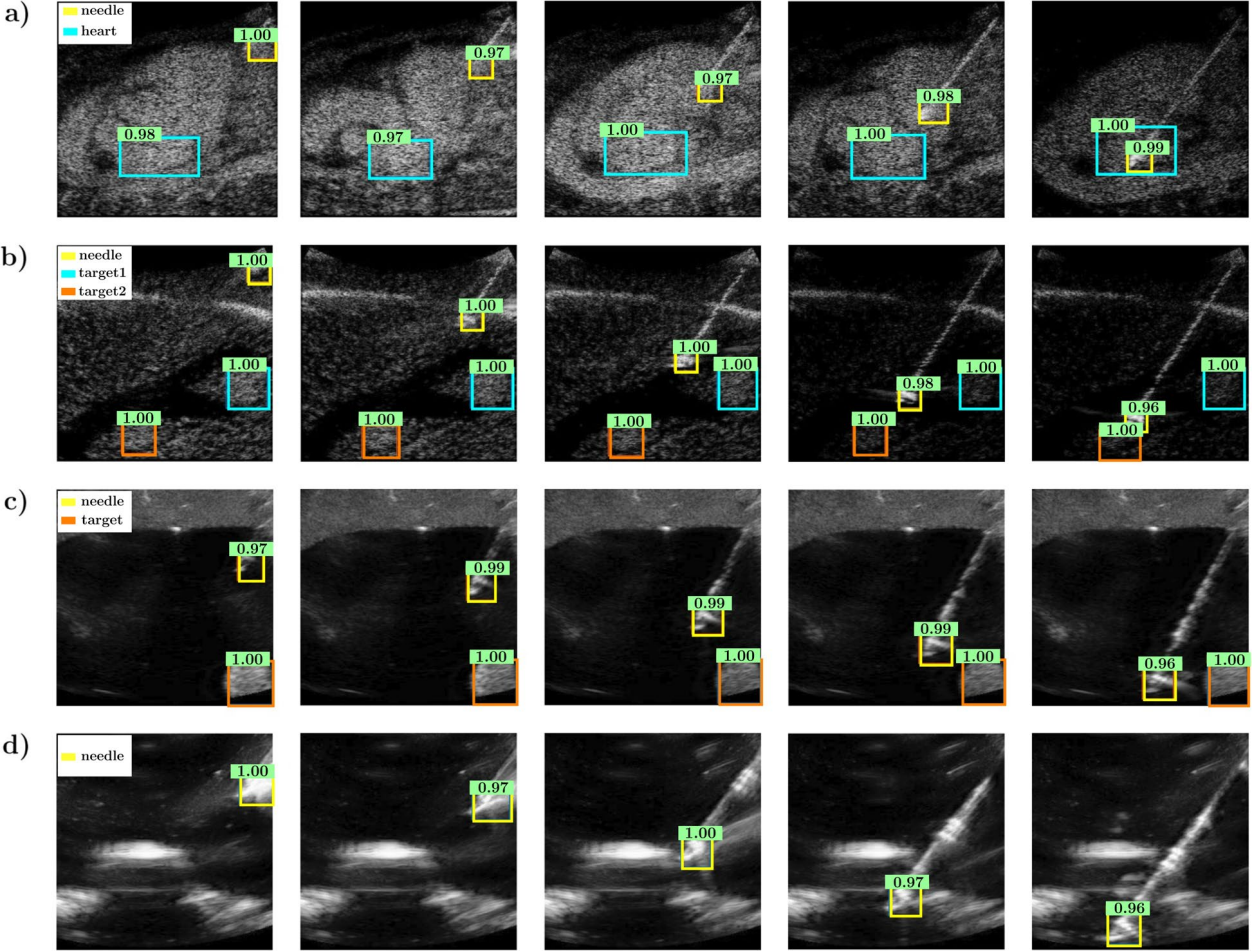
Evolution of needle and target detection in five different frames relative to the four testing datasets: synthetic data generated from magnetic resonance imaging scans, the target being the left atrium (**a**); synthetic data generated from the computed tomography scan, the targets being the two liver lesions (**b**); *in vitro* acquisition adopting the three-dimensional (3D) CIRS phantom with the liver lesion, with a different plane than the actual experiment in **b** used for the acquisition (**c**); and *in vitro* acquisition adopting the 3D-printed phantom filled with gelatin. In the last experiment, only the needle is the object to be localized in the ultrasound image. See the video (Supplemental Material) for more examples

Figure 5 shows the needle and target detection results of five salient frames from the four validation datasets each. Note that despite the presence of other high-intensity interfering artifacts in the B-mode data, the needle tip and the target were accurately localized. In particular, we can observe that for synthetic scenario#1 (Fig. 5a), the left atrium is well recognized, even though its irregular shape changes from frame to frame and despite the blurriness of its borders. In synthetic scenario #2 (Fig. 5b), the needle and the two lesions were successfully localized by the network. Figure 5c refers to *in vitro* acquisitions with a similar configuration as in synthetic scenario#2 as the same CIRS Phantom is used to perform the needle intervention. We can observe that the needle and the lesion are properly detected using the DETR previously trained with synthetic data. Finally, Fig. 5d shows the performance of the DETR for needle tip detection in *in vitro* US images of the 3D printed phantom filled with gelatin.

### “Robot in reach of target” results

We used the *in vitro* scenario#2 US images for the computation of the homography matrix *H* (six interventions) and its evaluation (three interventions). For the validation experiment, three needle insertions performed at different initial points and angles were performed. The estimated needle tip positions were obtained by projecting the MoCap coordinates into the 2D US space through the homography matrix *H*. The root mean square error of the projection expressed in the MoCap frame was 2.8 mm for all three evaluation insertions.

**Fig. 6:**
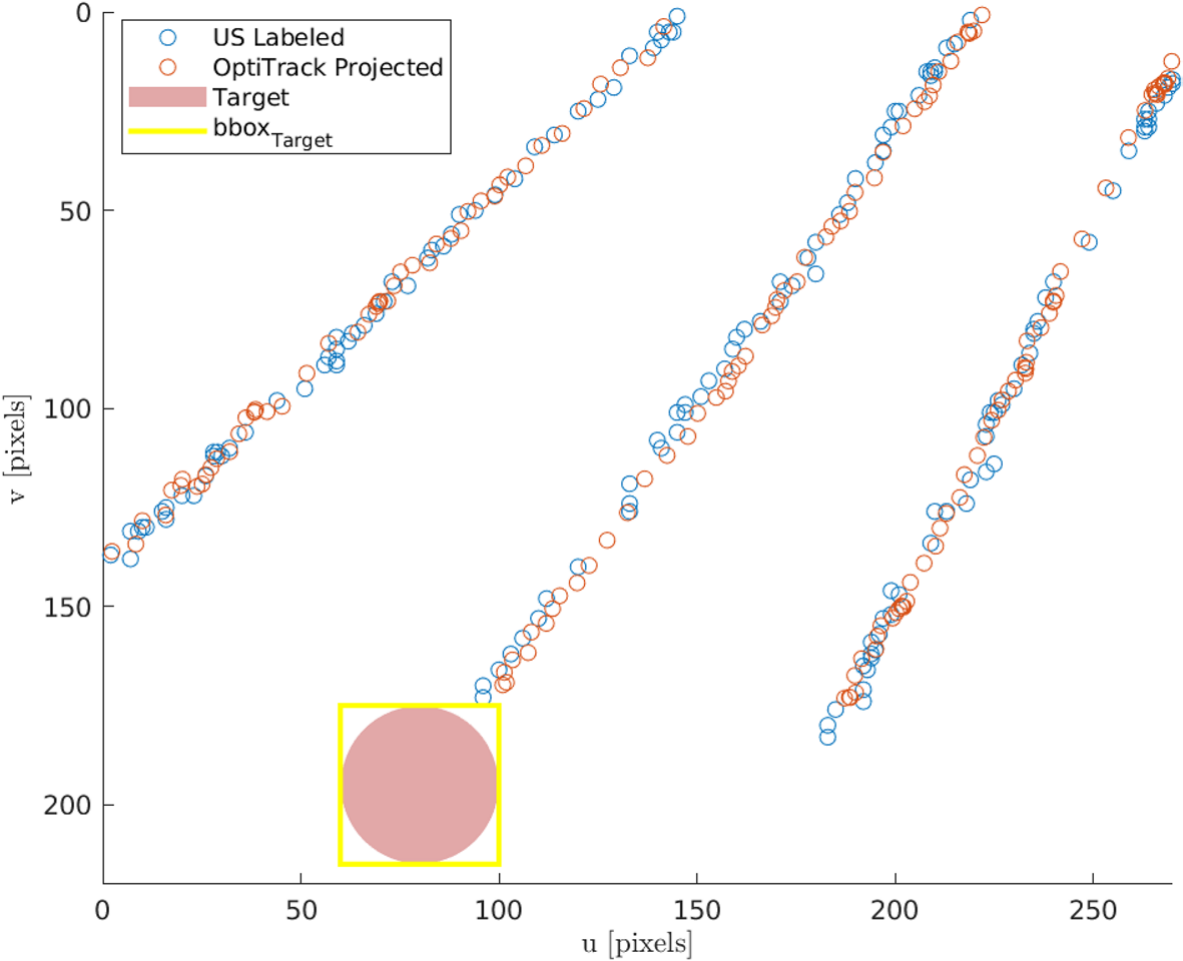
A validation example of the robot in reach method. Three different needle insertions at different initial points and angles are considered. The colorized blue and red circles represent the ground truth and the projected coordinates of the needle tip, respectively. The detected target through the detection transformer (DETR) is exploited to configure the robot positioning unit and the needle holder to perform a successful intervention

Figure 6 illustrates an example of the “robot in reach of target” method. If the target is detected in the US image by the DETR algorithm, its metric distance to a fixed reference point on the robot-US-scanner unit can be computed using the *H* matrix obtained in the calibration step. This enables one to position the robot such that the target can be reached with the needle. Figure 6 can be interpreted to show the possible trajectories of the needle tip in the US frame for different in-plane needle insertion angles prior to actual needle insertion. In this example, the center trajectory would successfully reach the desired target location. No MoCap system was needed for this; only the needle length and the available angles of the needle holder for needle insertion are sufficient to reconstruct the position of the needle tip, whereas state-of-the-art approaches require a MoCap system throughout the procedure.

## Discussion

A main challenge for DL-based algorithms is the need for large amounts of annotated data to train models of sufficient accuracy and robustness. While publicly available, annotated datasets for diagnostic imaging have been steadily increasing [29, 31, 32], datasets for interventional procedures are very limited and often are not annotated for target anatomy or surgical tools. To overcome this challenge, we have developed a novel simulation pipeline for the generation of clinical-like, interventional US images, including the necessary annotations for training state-of-the-art neural networks. Since 3D imaging datasets for diagnostic purposes such as CT or MRI are frequently available in clinical practice, including annotations of relevant regions of interest (*e.g.,* tumor lesions), the proposed US simulation pipeline can be used to generate training data for a variety of interventions, and even for patient-specific training data, without the need of time-consuming and error-prone manual annotation.

We have illustrated the validity of our approach by training DETR networks with synthetic data generated by our simulation pipeline using two different simulated clinical use cases and two different imaging modalities. We have observed that the needle detection performed very well in both the synthetic testing datasets (97% and 98% mAP, respectively). Also, the lesion detection performed very well (95% mAP), while the left atrium detection decreased (80% mAP) due to the fact that the latter is a very challenging task. Absent publicly available benchmark datasets, a direct comparison to state-of-the-art AI-based detection methods is difficult. Moreover, the approaches proposed in literature so far focused mostly on needle detection and not the more challenging needle and lesion/organ detection. The authors in [17] achieved an mAP of 95% with a frame rate of 10 frames/s, whereas we achieve an mAP of 98% for needle detection in both simulated scenarios and at 30 frames per second.

A crucial aspect in minimally invasive interventions is the initial positioning of the surgical tool with respect to the target. Different studies have reported promising CT image-guided navigation with C-arm systems combined with remote-operated positioning and guidance systems [33, 34], resulting, *e.g.,* in the reduction of radiation exposure while enhancing precision [34]. With cost efficiency and operational capacity in mind, a prototype robotic tool for US-guided biopsy during video-assisted surgery was proposed [35]. Under ideal conditions (target immersed in water), the system achieves a mean target localization error of 2.05 mm and a maximum error of 2.49 mm. In our work, we equipped the new version of the robotic platform introduced in [34] with a US scanner and have shown that our proposed method can be used to accurately position the micro-robot platform on the patient in order to be able to reach the target and based on 2D US images alone. The mean error we achieve (2.8 mm) is slightly bigger but comparable to that reported by other authors [35] even though our experiments were conducted using gelatin (and not water) which introduces some uncertainties due to needle bending induced by contact with a denser texture.

Even though the accuracy on the *in vitro* testing dataset is not as high as when evaluating with synthetic data (this is due to the very different clinical conditions as the network was trained on the synthetic heart dataset but deployed on the *in vitro* liver/abdomen dataset), the achieved performance is very good illustrating the sim-to-real capability of our data simulation and training approach. While we have validated our approach with *in vitro* data and feel confident that the results will translate to clinical data, this will be confirmed by future experiments. We have only evaluated the needle detection algorithm for situations where the needle is in-plane with the US imaging plane. Nevertheless, since the proposed method detects the needle tip (not the entire needle shaft), partially out-of-plane needle localization is still possible. We have observed in our experiments that the needle we have used is prone to slight bending. While this does not affect the accuracy of needle tip localization, it can have an adverse effect on the accuracy of the proposed “robot in reach of target method”. Needle localization accuracy with our algorithm was sometimes negatively affected if there are bright speckles in its vicinity.

We will further investigate these aspects in our future work to be able to draw conclusions for a wider array of different clinical situations. We have so far not investigated the impact of organ movement due to breathing or external pressure on our method. We will address this challenge in our future work by integrating the needle and target detection outcomes in a more complex state estimation structure relying also on the robotic platform.

## Data Availability

The datasets used and/or analyzed during the current study are available from the corresponding author on reasonable request.

## Abbreviations

2D: Two-dimensional
3D: Three-dimensional
CT: Computed tomography
DETR: Detection transformer
DL: Deep learning
mAP: Mean average precision
MoCap: Motion capture
MRI: Magnetic resonance imaging
Sim-to-real: Simulation-to-real
US: Ultrasound
