# Correction: Bridging the simulation-to-real gap for AI-based needle and target detection in robot-assisted ultrasound-guided interventions

**DOI:** 10.1186/s41747-023-00367-4

**Published:** 2023-07-19

**Authors:** Visar Arapi, Alexander Hardt-Stremayr, Stephan Weiss, Jan Steinbrener

**Affiliations:** Control of Networked Systems Research Group, Institute of Smart Systems Technologies, University of Klagenfurt, Klagenfurt, Austria

**Correction: Eur Radiol Exp 7, 30 (2023)**

**https://doi.org/10.1186/s41747-023-00344-x**

The production team handling the original article [1] erroneously typeset an incorrect image for Fig. 1. The correct image has since been re-instated.

**Fig. 1:**
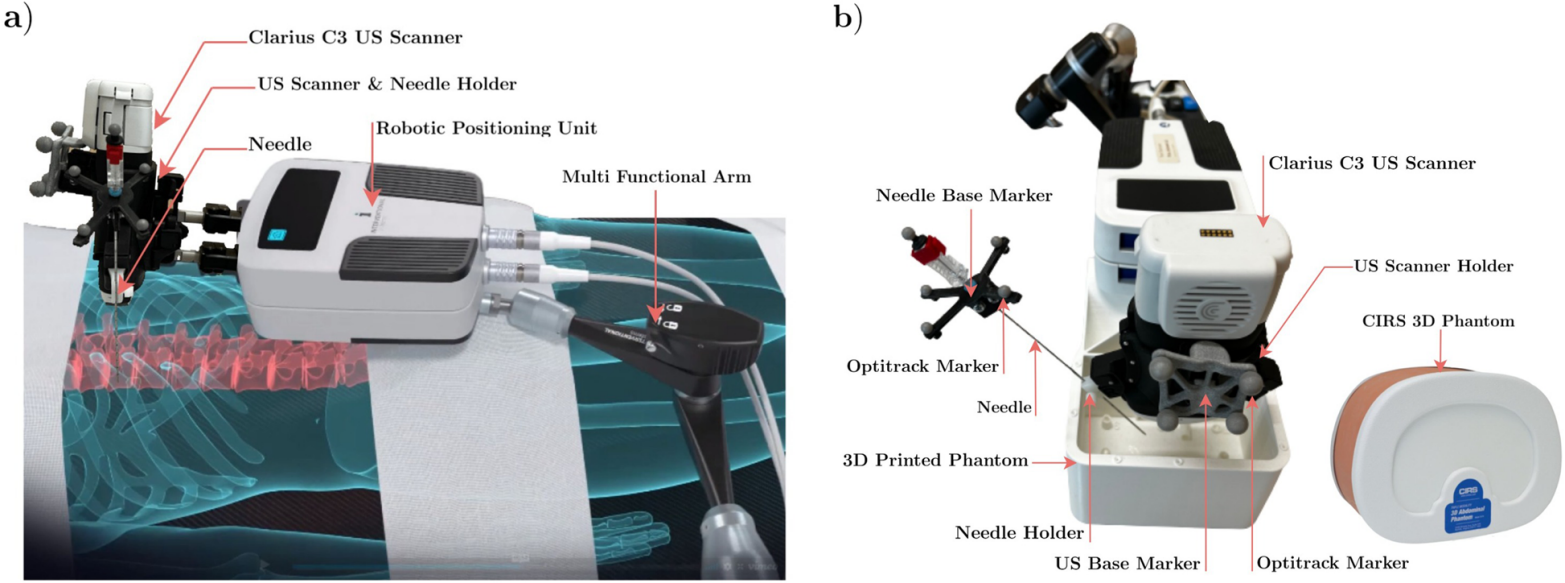
Interventional robotic system adopted in this work. **a** The ultrasound (US) scanner and the needle are mounted directly on the iSYS Micromate™ medical robotic platform through a specially designed holder so that the field of view of the US scanner can be controlled with the robot. **b** Experimental setup used for the in vitro US dataset collection. Reflective markers are attached via rigid bases to the US scanner and to the needle posterior allowing their tracking with the infrared camera system
